# Complications Following XEN45 Gel Stent and Glaucoma Drainage Device Implantation During Glaucoma Fellowship

**DOI:** 10.7759/cureus.65582

**Published:** 2024-07-28

**Authors:** P. Connor Lentz, Isabella V Wagner, Christian Draper, Bryan Ang, Nithya Boopathiraj, Darby Miller, Syril Dorairaj

**Affiliations:** 1 Ophthalmology, Mayo Clinic Alix School of Medicine, Jacksonville, USA; 2 Ophthalmology, Mayo Clinic, Jacksonville, USA; 3 Ophthalmology, Tan Tock Seng Hospital, Singapore, SGP

**Keywords:** glaucoma surgery, glaucoma, complication management, glaucoma drainage device, xen45 gel stent

## Abstract

Purpose

XEN45 Gel Stent and glaucoma drainage device (GDD) implantation is safe and effective for glaucoma treatment and should be taught during glaucoma fellowship training. However, complications may still occur, with potentially sight-threatening consequences. The purpose of this study is to describe the management of complications following a series of XEN45 Gel Stent and GDD surgeries performed over the course of glaucoma fellowship training.

Methods

This is a retrospective case series of XEN45 Gel Stent surgeries performed on 16 eyes and GDD surgeries performed on seven eyes. Patient demographics, disease characteristics, and complications are reviewed. The intra- and postoperative course of five select cases with complications are described in detail.

Results

The most frequent complications following XEN45 implantation were transient hypotony (10 eyes, 63%), reduced visual acuity (VA) (five eyes, 31%), choroidal effusion (three eyes, 19%), hyphema (two eyes, 13%), and intraocular pressure (IOP) spike (two eyes, 13%). Thirteen eyes (81%) required bleb needling, and three eyes (19%) required XEN45 replacement. Complications following GDD implantation included hypotony (three eyes, 43%), reduced VA (two eyes, 29%), choroidal effusion (two eyes, 29%), IOP spike (two eyes, 29%), implant exposure (two eyes, 29%), and shallow anterior chamber (one eye, 14%). Three eyes (43%) required revision or explantation with a secondary glaucoma surgery. One choroidal effusion following XEN45 surgery and one following GDD surgery were hemorrhagic choroidal effusions requiring surgical drainage.

Conclusion

Significant and potentially sight-threatening complications may occur following XEN45 Gel Stent and GDD implantation performed over the course of fellowship training. Glaucoma fellows should be ably equipped to recognize, diagnose, and manage these complications both intra- and postoperatively.

## Introduction

Surgical treatment options for glaucoma have evolved significantly in recent years. Traditionally, trabeculectomy has been considered the “gold standard” for surgical management when other first-line interventions, such as medications and lasers, prove insufficient to slow disease progression. Trabeculectomy involves the surgical creation of a drainage channel to provide an alternative aqueous humor outflow pathway. Although largely safe and effective in reducing intraocular pressure (IOP) and glaucomatous progression [1], it may be occasionally associated with significant perioperative complications [2,3]. For patients with a higher risk of trabeculectomy failure or with complex forms of glaucoma, glaucoma drainage device (GDD) implantation has emerged as a successful treatment alternative [4-6]. First developed in the 1960s, GDDs have undergone various modifications designed to improve long-term efficacy and safety. However, GDDs are nonetheless still associated with several intra- and postoperative complications [7].

In recent years, the advent of minimally invasive glaucoma surgery (MIGS) has significantly expanded the glaucoma surgical armamentarium. MIGSs are increasingly being used as earlier interventions for glaucoma and have begun to replace traditional surgical methods in some clinical situations. The XEN45 Gel Stent (AbbVie, Chicago, IL) is a minimally invasive stent that functions similarly to trabeculectomy and GDDs in that it redirects aqueous humor to the subconjunctival space and forms a filtering bleb. Primarily indicated for patients with primary open-angle glaucoma (POAG), the XEN45’s smaller size and less invasive surgical approach theoretically reduce complications associated with traditional filtering glaucoma procedures. However, various complications following XEN45 implantation have been reported throughout the literature [8,9].

In this retrospective case series, we report the safety profile and complications associated with both XEN45 and GDD implantation performed by a glaucoma fellow, over the course of fellowship training. We further describe the subsequent clinical and surgical management of complications occurring in five selected cases.

## Materials and methods

In this retrospective case series, we report complications following XEN45 and GDD implantation performed by a glaucoma fellow over the course of a one-year fellowship. The study was reviewed and approved by the Mayo Clinic Institutional Review Board (IRB) and was conducted in accordance with the tenets of the Declaration of Helsinki. The inclusion criteria consisted of adult eyes that underwent XEN45 or GDD implantation between 8 January 2022 and 5 January 2023. All surgeries were performed by the same glaucoma fellow under faculty supervision at a single tertiary care center unless otherwise specified. Demographics and complications were reported for all patients. 

Surgical technique: XEN45 Gel Stent

Ab-interno or ab-externo approaches were utilized for XEN45 implantation across all cases. Ab-interno XEN45 placement involved the creation of a paracentesis and viscoelastic filling of the anterior chamber, followed by the insertion of the XEN45 at the iridocorneal angle of the 12 o’clock position, emerging into the subconjunctival space approximately 2.5 mm posterior to the superior limbus. Mitomycin C (MMC) 20 mcg (200 mcg/mL) was subsequently injected in the subconjunctival space and primary needling was performed, where a needle was used to ensure the tip of the stent was free and mobile following implantation. In ab-externo stent placement, the stent was implanted following an open conjunctival or transconjunctival approach. The open conjunctival approach involved the creation of a superior limbal peritomy and subsequent dissection to separate the conjunctiva and Tenon’s capsule away from the sclera. MMC 20 mcg (200 mcg/mL) was either injected or applied by a soaked sponge and washed thoroughly with balanced salt saline before stent implantation through a scleral tunnel 2.5 mm posterior to the limbus, into the anterior chamber. The transconjunctival approach involved inserting the stent through closed conjunctiva 7 mm posterior to the superior limbus and a scleral tunnel 2.5 mm posterior to the limbus to enter the anterior chamber. Placement of the stent was confirmed with intraoperative gonioscopy. MMC was then injected into the subconjunctival space and primary needling was performed.

Surgical technique: GDDs 

The GDDs used were the Ahmed FP-7 valved device (New World Medical, Rancho Cucamonga, CA) and the Ahmed ClearPath non-valved device (New World Medical, Rancho Cucamonga, CA). 

For Ahmed FP-7 implantation, conjunctiva and Tenon’s capsule were first incised at the limbus and dissected to create a fornix-based peritomy in the superotemporal quadrant. The tube was gently primed with balanced salt saline. The plate was secured to the sclera using a nylon suture with the anterior plate edge approximately 8 mm posterior to the limbus. A 23-gauge needle was used to create a sclerostomy into the anterior chamber. The tube was trimmed to the appropriate length and inserted through a scleral tunnel 1 mm from the limbus at the 12 o’clock position into the anterior chamber. The tube was positioned in an “S” configuration against the sclera to accommodate plate movement without affecting the tube’s position in the anterior chamber and then secured to the sclera using a nylon suture. The plate and the tube were then covered with a pericardial patch graft and secured with polyglactin suture and fibrin glue before the conjunctiva and Tenon’s capsule were closed. 

For implantation of the Ahmed ClearPath, conjunctiva and Tenon’s capsule were incised at the limbus and dissected to create a fornix-based peritomy in the superotemporal quadrant. The plate was secured to the sclera using a nylon suture with the anterior plate approximately 7 mm posterior to the limbus. The tube was ligated with polyglactin suture and adequate obstruction to flow through the tube was confirmed by attempted injection of balanced saline. A 23-gauge needle was used to create a sclerostomy into the anterior chamber. The tube was trimmed to the appropriate length and inserted through a scleral tunnel 1 mm from the limbus at the 12 o’clock position into the anterior chamber. Two venting fenestrations were created in the tube using a suture needle and then secured to the sclera using a nylon suture. The polypropylene ripcord was then tucked under the inferior conjunctiva. The plate and the tube were then covered with a pericardial patch graft and secured with polyglactin suture and fibrin glue before the conjunctiva and Tenon’s capsule were closed.

## Results

Five complicated cases and their subsequent management are described in detail. 

XEN45 Gel Stent

XEN45 implantation was performed on 16 eyes of 14 patients over the course of the glaucoma fellowship. The mean duration of patient follow-up was 9.8 months, with the shortest follow-up period being 5.0 months and the longest extending to 14.0 months post-surgery. Table 1 shows demographics and baseline characteristics.

**Table 1:** XEN Gel Stent Patients: Demographics and Baseline Characteristics

XEN Gel Stent	Total Number, n (%)
Number of Patients	14
Number of Eyes	16 (100)
Right Eyes	6 (38)
Left Eyes	10 (63)
Age (Years)	
Mean (Standard Deviation)	78 (13)
Median	67
Range	33-87
Sex	
Male	9 (56)
Female	7 (44)
Race	
White	13 (81)
Indian	2 (13)
Hispanic	1 (6)
Diagnosis	
Primary Open-Angle Glaucoma	12 (75)
Pseudoexfoliative Glaucoma	1 (6)
Pigmentary	1 (6)
Chronic Angle Closure	1 (6)
Ocular Hypertension	1 (6)
Severity	
Mild	1 (6)
Moderate	2 (13)
Severe	11 (69)
Indeterminate	2 (13)
Surgical Approach	
Ab-interno	2 (13)
Ab-externo (Open Conjunctiva)	2 (13)
Ab-externo (Transconjunctival)	12 (75)

The mean age of these patients was 78 (13) years, and 13 of 16 eyes (81%) were from White patients. Twelve of 16 eyes (75%) had severe POAG. A transconjunctival approach was the preferred technique for 12 of 16 eyes (75%).

Complications are reported in Table 2.

**Table 2:** XEN Complications (N=16)

XEN Gel Stent	Total Number, n (%)
Required Revision	4 (25)
Needling x1	3 (19)
Needling x2	7 (44)
Needling x3 or more	4 (25)
Required Secondary Glaucoma Surgery	3 (19)
Reduced Visual Acuity by More Than Two Lines at 3 Months Post-op	5 (31)
Hypotony	10 (63)
Choroidal Effusion	3 (19)
Hyphema	2 (13)
Intraocular Pressure Spike	2 (13)
Implant Exposure	0
Shallow Anterior Chamber	0
Bleb Leak	0
Infection	0

Revision refers to stent explantation, new XEN45 implantation, or opening of the conjunctiva to explore stent patency. Indications for postoperative needling included elevated IOP or bleb vascularization noted on slit lamp examination throughout the first six weeks following implantation. Needling was typically performed in the outpatient setting, accompanied by an injection of 5-fluorouracil (5-FU) 7.5 mg (50 mg/mL) into the bleb. MMC was used in cases where needling was performed in the operating room, in combination with a new XEN implantation or with other incisional surgery. Three patients underwent XEN stent explantation with secondary stent implantation due to failure of the primary XEN stent.

Reduced visual acuity (VA) was defined as the loss of more than two lines of best-corrected vision on the Snellen’s acuity chart. One patient had reduced VA due to choroidal hemorrhage resulting from the surgery. Two patients had reduced VA due to corneal disease progression (superficial punctate keratitis and keratouveitis) unrelated to XEN surgery, one patient had cystoid macular edema at the three-month follow-up unrelated to the XEN implantation, and one patient had intraocular lens (IOL) subluxation also unrelated to the XEN implantation. 

The most common postoperative complication was hypotony, which was defined as an IOP of ≤5 mmHg occurring postoperatively or post-needling. Transient hypotony occurred in 10 of 16 eyes but all cases resolved by month 1 post-procedure and there were no cases of persistent hypotony. Choroidal effusions occurred in three eyes. One case occurred with hypotony on postoperative day (POD) 4 and resolved without surgical intervention. Another case occurred without hypotony and resolved spontaneously as well. The final case was associated with hypotony and resulted in large hemorrhagic effusions which required subsequent drainage. Hyphema occurred in one patient at postoperative week (POW) 1 and the other occurred shortly after revision with a new XEN stent. The hyphema in both cases resolved spontaneously. IOP spike was defined as having an IOP above 30 mmHg or 10 mmHg above the baseline IOP. One patient had an IOP spike due to a steroid response. The other case of IOP spike resulted from the hemorrhagic choroidal effusion mentioned previously. There were no cases of implant exposure, shallow anterior chamber, bleb leak, or infection. 

Glaucoma drainage devices

GDDs were implanted in seven eyes of seven patients from November 2022 until April 2023. The mean duration of patient follow-up was 9.4 months, with a minimum of 7.0 months and a maximum of 12 months. Demographics and baseline characteristics are shown in Table 3.

**Table 3:** Glaucoma Drainage Device Patients: Demographics and Baseline Characteristics

Glaucoma Drainage Devices	Total Number, n (%)
Number of Patients	7
Number of Eyes	7 (100)
Right Eyes	1 (14)
Left Eyes	6 (86)
Age (years)	
Mean (Standard Deviation)	75 (10)
Median	72
Range	63-88
Sex	
Male	4 (57)
Female	3 (43)
Race	
White	5 (71)
Black	1 (14)
Hispanic	1 (14)
Diagnosis	
Primary Open-Angle Glaucoma	3 (43)
Pseudoexfoliative Glaucoma	1 (14)
Neovascular	2 (29)
Uveitic	1 (14)
Severity	
Mild	0 (0)
Moderate	1 (14)
Severe	6 (86)
Indeterminate	
Device Type	
Ahmed FP-7	6 (86)
Ahmed ClearPath	1 (14)

The mean age (standard deviation) of these patients was 75 (10) years. Five of seven (71%) eyes were from Caucasian subjects. Three of seven (43%) eyes had POAG, two (29%) eyes had neovascular glaucoma, one (14%) eye had uveitic glaucoma, and one (14%) eye had pseudoexfoliative (PEX) glaucoma. Six of seven eyes were diagnosed with severe, refractory glaucoma. Of the seven GDDs implanted, six were the Ahmed FP-7 valved device and one was an Ahmed ClearPath. 

 Table 4 shows the complications resulting from GDD implantations.

**Table 4:** Glaucoma Drainage Device Complications (N=7)

Glaucoma Drainage Devices	Total Number, n (%)
Required Revision	3 (43)
Required Secondary Glaucoma Surgery	4 (57)
Reduced Visual acuity by More Than Two Lines at 3 Months Post-op	2 (29)
Hypotony	3 (43)
Choroidal Effusion	2 (29)
Hyphema	0
Intraocular Pressure Spike	2 (29)
Implant Exposure	2 (29)
Shallow Anterior Chamber	1 (14)
Bleb Leak	0
Corneal Decompensation	0
Infection	0

Three of seven GDDs required revision followed by secondary glaucoma surgery, and in two of these cases, the plate or tube became exposed and required explanation. In one case, the tube of the device was noted postoperatively to be too long and required tube trimming and IOL exchange. One patient underwent MicroPulse transscleral cyclophotocoagulation (MP-TSCPC) for uncontrolled IOP without revision of the drainage device. Reduced VA by more than two lines was reported in two patients at postoperative month (POM) 3. One case was due to lens subluxation and the other from myopic degeneration and macular scarring from prior ocular histoplasmosis.

IOP spikes occurred in two patients at the time of tube erosion and after tube explantation, respectively. Hypotony was noted in three patients - two cases occurred shortly after surgery and one case occurred after explantation of the GDD. This same GDD explantation also resulted in choroidal effusion that resolved spontaneously. A second case with choroidal effusion was a hemorrhagic effusion that required surgical drainage. Nearly all patients had a history of multiple previous ocular surgeries including XEN implantation, goniotomy, canaloplasty, and transscleral cyclophotocoagulation that likely contributed to the complications observed. Several of these patients are detailed below.

Case 1

This patient was a 77-year-old male with an ocular history of severe bilateral POAG with multiple failed glaucoma surgeries in his left eye. He had previously undergone cataract extraction, goniotomy, canaloplasty, and 2 MP-TSCPC procedures. Preoperatively, the patient was on four IOP-lowering medications (brimonidine 0.2%, brinzolamide 1%, latanoprostene bunod 0.024%, and latanoprost 0.005%). The patient’s right eye IOP was moderately controlled with surgery and medications, but his left eye IOP remained at 35 mmHg one year after goniotomy and canaloplasty. 

**Figure 1:**
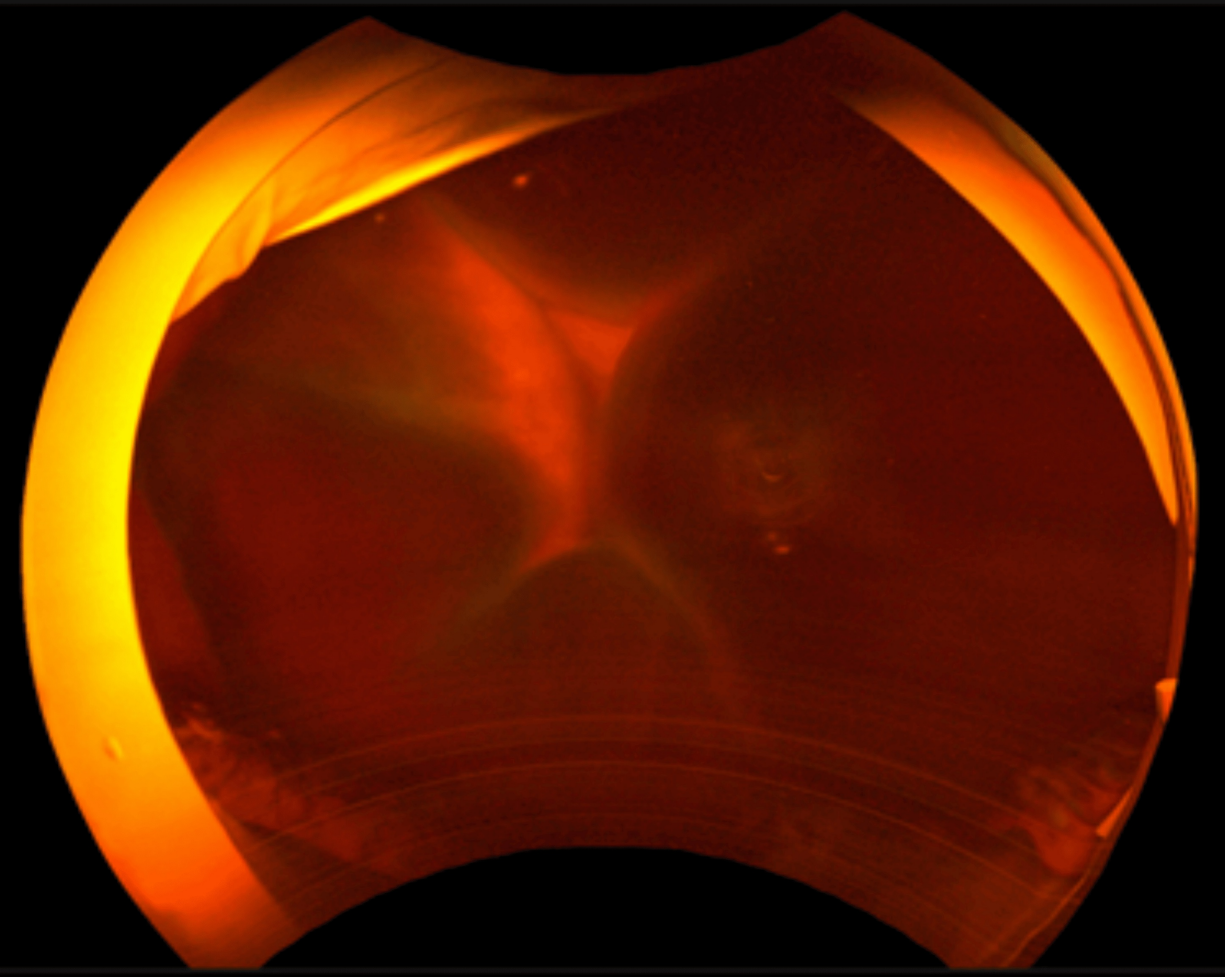
Large serous choroidal effusions seen post-XEN45 implantation in case 1.

A XEN45 was implanted into the left eye through an ab-externo transconjunctival approach with MMC augmentation and the surgery was uneventful. However, the IOP was 3 mmHg at POD 1. At POD 4, the patient complained of severe left-sided headache and eye pain with nausea. Upon examination, large serous choroidal effusions were seen in the left eye, with the IOP at 4 mmHg (see Figure 1).

Atropine 1% was started and one day later, the IOP spiked to 51 mmHg. Glaucoma medications were restarted immediately, with partial IOP reduction.

The decision was made to surgically drain the hemorrhagic choroidal effusions three weeks post-XEN45 implantation after allowing for liquefaction to occur. A retina specialist performed the procedure with the glaucoma fellow assisting. Postoperatively the IOP was 18 mmHg in the left eye. Hypotony returned immediately following the drainage procedure but both hypotony and eye pain resolved two months after surgery. VA was 20/25 before XEN45 implantation and decreased to light perception (LP) for over one month following implantation. Although the prognosis was guarded following the significant complications experienced by this patient, the VA eventually improved to 20/40 at the most recent follow-up nine months post-XEN45 implantation.

Case 2

This patient is an 88-year-old female with an ocular history of severe bilateral PEX glaucoma with refractory high IOP in her left eye. The patient was on three medications (brimonidine 0.2%, latanoprost 0.005%, and netarsudil 0.02%). The patient had previously undergone canaloplasty and MP-TSCPC. However, two months after the MicroPulse laser, the left eye IOP was noted to be 43 mmHg and the best-corrected VA was 20/50. A decision was made to proceed with Ahmed FP-7 implantation.

The patient had no intraoperative complications. The left eye IOP was initially well-controlled but began to rise to low 20s mmHg at POM 3. At POM 4, the patient returned to the clinic for continuous blurry vision. She was found on the exam to have a new-onset vitreous hemorrhage and subluxated lens, resulting in a decreased VA of 20/100. Together with the vitreoretinal specialist, the collective decision was made to control the IOP before performing lens replacement and vitrectomy.

A goniotomy, in combination with canaloplasty, was performed for the patient. Intraoperatively, it was observed that the tube of the Ahmed FP-7 was in contact with and pushing against the patient’s IOL. In the setting of weak zonules from pseudoexfoliation, it was suspected that this had led to the subluxation of the IOL. The tube was then trimmed to an appropriate length, and the bleb was needled with a 30-gauge needle, with MMC augmentation. Two months after this procedure, an anterior vitrectomy with secondary IOL fixation was performed, again together with a bleb needling with a 30-gauge needle and MMC augmentation. Ten months following the initial Ahmed FP-7 implantation, the patient’s left eye IOP remained stable in the range of mid-teens mmHg and VA improved to 20/60.

Case 3

This patient was a 72-year-old male with an ocular history of bilateral severe POAG. At presentation, the patient was on three glaucoma medications (latanoprost 0.005%, dorzolamide 2%, and betaxolol 0.25%). Despite previously undergoing selective laser trabeculoplasty (SLT), phacoemulsification with trabeculotomy, MP-TSCPC, and XEN45 implantation, the left eye IOP slowly rose to be in the mid-20s mmHg at 1.5 years post-XEN45 implantation. A decision was made to implant an Ahmed FP-7 Valve with a pericardial patch graft. The flat bleb from the prior XEN stent was also needled at this time with needling and MMC augmentation.

Postoperatively, the left eye was hypotonous (IOP of 3-4 mmHg) for one month with distance VA decreasing to 20/50, from a baseline of 20/25. At POM 2, the patient presented with redness, swelling, and pain in the left eye. Upon examination, the Ahmed FP-7 tube had become exposed and the IOP was elevated at 32 mmHg. The patient underwent surgical revision of the exposed tube, with pericardium used to cover the eroded portion of the tube, followed by conjunctival closure and amniotic membrane graft placement with absorbable suture and fibrin glue to cover any areas of inadequate closure. Following the surgery, the patient’s left eye once again was noted to be hypotonous (IOP of 5 mmHg) which persisted for about one month.

One month after the placement of the patch graft, the tube was noted to be exposed once again. A decision was made to explant the GDD tube and implant a new XEN45 Gel Stent in the left eye. The previous XEN45 was extracted, and the new stent was inserted using an ab-externo transconjunctival approach. Primary needling was performed, and MMC was injected and irrigated. At POD 1, transient hypotony was noted but the IOP returned to a normal range at POW 1. IOP has remained stable, and VA recovered to 20/25 at the last follow-up 11 months after the initial Ahmed FP-7 surgery.

Case 4

This patient was a 65-year-old male with an ocular history of bilateral, refractory severe POAG, bilateral ocular histoplasmosis resulting in macular scarring, and bilateral myopic degeneration. He had previously undergone SLT, phacoemulsification with goniotomy, XEN45 Gel Stent implantation, and MP-TSCPC three times in his left eye. At presentation, the patient was using four glaucoma medications (latanoprost 0.005%, brimonidine 0.2%, and dorzolamide 2%-timolol 0.5%). IOP in the left eye was elevated to 29 mmHg nine months after his last MicroPulse intervention, and a decision was made to implant an Ahmed FP-7 Valve. 

**Figure 2:**
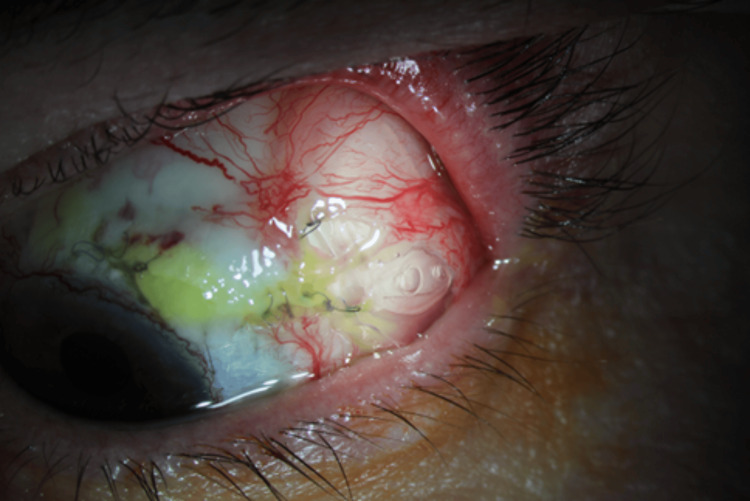
Exposed Ahmed FP-7 Valve plate seen six months after a complicated implantation in case 4.

The Ahmed FP-7 was implanted and covered with processed pericardium and an amniotic membrane graft. Due to the patient’s extensive surgical history, the conjunctiva had significant adhesions and was further complicated by intraoperative subconjunctival hemorrhage. Intraoperatively, the bleb from the previous XEN Gel Stent was needled with MMC injection and irrigation. At the six-week postoperative visit, it was noted that the plate of the drainage device was becoming exposed (see Figure 2).

The patient was taken back to the operating room and the conjunctiva around the plate was dissected as much as possible in the setting of conjunctival scarring, and again, processed pericardium and amniotic membrane were used to cover the exposed plate. Within two weeks, the plate became exposed again, so the decision was made to explant the Ahmed FP-7 and cover the defect with processed pericardium and an amniotic membrane graft. Following explantation, hypotony occurred on POD 1 and 2. The patient returned to the clinic on POD 5 with left eye pain, blurred vision, and an IOP of 55 mmHg. The patient was seen again the next day and was found to have an unchanged IOP with hyphema. 

The following morning, the patient was taken to the operating room for wound revision and anterior chamber washout. The patch graft was removed and scarred conjunctiva was trimmed around the wound edges. Extensive conjunctival dissection was performed, and a sliding conjunctival autograft was mobilized to cover the defect. At this time, a goniotomy was also performed. 

The left eye IOP lowered to the mid-20s mmHg after this surgery, but quickly rebounded to above 30 mmHg, where it persisted for one month on topical medications until a new XEN45 Gel Stent was implanted. The XEN45 Gel Stent was implanted with an ab-externo transconjunctival approach and primary needling of the bleb was performed with MMC injection and irrigation. Postoperatively, IOP remained stable at a normal range. Choroidal effusions were noted but resolved spontaneously after one week. Bleb needling with 5-FU injection was performed in the office on two separate occasions, at one week and one-month post-XEN implant. The IOP remained stable at the most recent visit six months post-XEN implant. VA was 20/80 at baseline before Ahmed FP-7 implantation and 20/200 at the most recent visit at one year following the initial Ahmed FP-7 implantation.

Case 5

This patient was an 82-year-old male with type 2 diabetes mellitus, with an ocular history of severe POAG and, most recently, neovascular glaucoma in his left eye. The patient initially presented two years prior with sudden vision loss in his left eye with a VA of 20/80, which was determined to be resulting from severe POAG and atypical non-arteritic anterior ischemic optic neuropathy. Interval surgical history included XEN45 Gel Stent implantation on two separate occasions, with bleb revisions, and MP-TSCPC. Previous postoperative complications included hyphema and choroidal effusions that resolved spontaneously. The patient later suffered a central retinal vein occlusion, and over the course of two years, VA decreased from a baseline of 20/80 to LP. One year later, the patient presented with acute left eye pain, redness, and hyphema with an IOP of 45 mmHg, and was diagnosed with neovascular glaucoma. IOP did not improve despite anti-vascular endothelial growth factor therapy, or medical therapy with topical brimonidine 0.1%, dorzolamide-timolol 2-0.5%, or oral methazolamide 100 mg daily.

An Ahmed ClearPath device was implanted uneventfully, and the processed pericardium was used to cover the tube. On POD 1, the patient was noted to have a shallow anterior chamber and left eye IOP of 35 mmHg. At POW 1, the IOP dropped to 10 mmHg, and hemorrhagic choroidal effusions were found. The patient developed significant pain in his left eye over the following week and large “kissing” choroidal effusions were seen on B-scan ultrasound. The decision was made to surgically drain the choroidal hemorrhages three weeks post-implantation of the GDD. An anterior chamber maintainer was placed, conjunctival peritomy was performed, and drainage sclerotomies were created nasally and temporally. Minimal serous fluid and copious old blood were removed through passive expression. The following day, a B-scan showed the choroidal effusions had resolved. Following the procedure, the patient’s IOP remained stable in the normal range and the eye pain completely resolved. The patient had a recurrence of choroidal effusions two months post-drainage, but this time achieved resolution with conservative management and topical medications (atropine 1%).

## Discussion

Fourteen of 16 eyes with XEN45 implantation had at least one complication postoperatively. Transient hypotony was the most common complication, occurring in 71% of eyes. Nearly all resolved spontaneously by POM 1 and were not clinically significant. Hyphema and IOP spike occurred in 14% and 21% of eyes, respectively. The only complications that required interventions were choroidal effusions in two eyes, one of which was treated with atropine 1% and the other requiring surgical drainage as described above. There were no instances of endophthalmitis, bleb leak, or implant exposure. 

Systematic reviews and meta-analyses of XEN45 Gel Stent surgery outcomes have consistently showed hypotony as the most common complication after XEN implantation, occurring after 10-39% of surgeries [10-12]. Hyphema is frequently cited as the second most common complication, occurring after 4-14% of surgeries. IOP spikes occurred in 2-13% of cases. Other complications, including choroidal effusions and stent exposure, were rare and occurred in 1% or less of XEN patients.

Though our patients experienced higher rates of hypotony than averages reported in prior systematic reviews, a paper by Post et al. with a similarly small sample size reported as many as 90% of their cases had transient hypotony within the first week [13]. Other studies have also reported a similar percentage of patients experiencing IOP spikes in the first year after XEN surgery [9]. The small sample size, complex patient population, and limited experience of the glaucoma fellow are the most likely contributing factors influencing the rates of some complications seen in these patients.

Six out of the seven GDDs implanted were Ahmed FP-7 valves. The unique design of the Ahmed FP-7 is intended to limit IOP lowering and prevent hypotony [7]. Previous comparison studies between GDDs show that Ahmed FP-7 valves have reduced but not eliminated instances of hypotony [14-16]. Only one of our Ahmed FP-7 patients experienced hypotony immediately after surgery, which lasted for one month. Another Ahmed FP-7 patient experienced hypotony and choroidal effusion after explantation of the device.

The Ahmed ClearPath is a newer, valveless GDD with a refined plate and low profile that conforms to the curvature of the eye. Consistent with its valveless nature, a previous study has shown early hypotony occurring in 7% of cases with choroidal effusions occurring in 2% of cases [17]. To prevent such complications, a Vicryl tie suture may be used to restrict flow through the tube. The 4-0 polypropylene ripcord prepackaged with the device may also be left attached to decrease the risk of hypotony [18]. Despite performing these measures in the patient outlined in case 5, the ClearPath implantation resulted in hypotony, shallow anterior chamber, and hemorrhagic choroidal effusion that required treatment with surgical drainage. 

Most choroidal effusions seen in our patients were benign and resolved spontaneously, but two required surgical intervention. Choroidal effusions are differentiated as either serous or hemorrhagic. Serous effusions typically involve serum accumulating in the suprachoroidal space following a decrease in IOP. Thus, hypotony following glaucoma surgery is the most common risk factor for serous effusions [19]. Hemorrhagic choroidal effusions are caused by blood accumulating in the suprachoroidal space. When there is a major and sudden reduction in IOP, posterior ciliary arteries may rupture and allow blood to enter this space. 

Though GDDs have grown in popularity partly due to a superior complications profile to trabeculectomy, it has been argued that postoperative suprachoroidal hemorrhages may occur more often following GDD implantation [20-22]. One possible explanation for this is that GDDs are often implanted in patients with higher baseline IOPs and the resulting drop in IOP is more drastic than in trabeculectomy. Likewise, the XEN45 Gel Stent is expected to decrease complications seen with GDDs and trabeculectomy due to its minimally invasive properties, but surgeons have reported that hemorrhagic choroidal effusions still occur [23,24]. 

Other risk factors for choroidal effusion are older age, pseudophakia, high myopia, diabetes mellitus, and hypertension [19,25,26]. Certain types of glaucoma have also been found to be risk factors, such as PEX glaucoma and neovascular glaucoma [19]. Systemic risk factors include cardiac disease, respiratory disease, and preoperative anticoagulation [21,27]. 

Both patients who required surgical drainage had several risk factors for hemorrhagic choroidal effusion. They were both of older age, pseudophakic, and had a history of multiple glaucoma surgeries. They also had a systemic history of hypertension, diabetes, and cardiac disease. One patient had a history of lung transplant, and the other had neovascular glaucoma. Possibly the main contributor to the hemorrhagic effusion was that both patients were taking anticoagulants preoperatively. Though they were both advised to discontinue anticoagulants prior to their respective surgeries, only one patient discontinued anticoagulants as recommended.

Conservative management of choroidal effusions is often preferred when the choroidal effusion is associated with hypotony. Medications like atropine and topical steroids can be given for persistent effusions. If medications fail, surgical drainage is the preferred treatment method. Other major indications for surgical drainage include a flat anterior chamber, significant eye pain, hemorrhagic choroidal effusion, and decreased vision [20]. 

In a study of choroidal effusion surgical drainage outcomes, WuDunn et al. reported that 77% of 63 eyes experienced surgical success by 12 months post-drainage [28]. Ninety percent of successful drainages achieved resolution of the choroidal effusions by POW 6, and most patients also achieved improved VA and hypotony resolution as well. In some cases, multiple drainages were required. Both of our patients achieved resolution of hypotony and eye pain, and one had improved VA. One patient had a complete resolution of choroidal effusions, but the other had a recurrence two months after the procedure following the restarting of anticoagulants. 

Although hemorrhagic choroidal effusions are rare, they are linked to considerable morbidity. When cases cannot be effectively managed with medications alone, surgical drainage can serve as an effective treatment option. Many glaucoma surgeons have limited experience performing choroidal effusion drainages and consequently, it may not be necessary for glaucoma specialists to develop proficiency in performing surgical drainage. Nonetheless, we strongly advise glaucoma surgeons to develop familiarity with complications associated with bleb-forming procedures and their appropriate management. Limitations of this study include its small sample size and short follow-up time. 

## Conclusions

The rapidly increasing number of glaucoma interventions presents a significant challenge for glaucoma surgeons to attain proficiency in all surgical approaches. XEN45 Gel Stents and GDDs are commonly employed in the management of refractory glaucoma patients, but both carry the risk of substantial and potentially sight-threatening complications. This risk is escalated in complex patients with a history of multiple glaucoma surgeries and concurrent systemic conditions. Cases involving the management of postoperative complications in such patients are described in this review. It is imperative for glaucoma surgeons not only to gain expertise in these surgical procedures but also to develop familiarity with the approaches that are required to manage complications arising from these procedures.
